# Estimated End-Tidal Sevoflurane Concentration to Maintain Optimal Anesthetic Depth During Cardiopulmonary Bypass: A Meta-Analysis

**DOI:** 10.3390/biomedicines14030535

**Published:** 2026-02-27

**Authors:** Sou-Hyun Lee, Tae Hoon Kang, Sungah Yoo, Kyungmi Kim

**Affiliations:** 1 Department of Anesthesiology and Pain Medicine, Kyungpook National Medical Center, Kyungpook National University School of Medicine, Daegu 41944, Republic of Korea; youlion6@gmail.com (S.-H.L.); thkang0118@naver.com (T.H.K.); 2 Department of Anesthesiology and Pain Medicine, Anam Hospital, Korea University College of Medicine, Seoul 02841, Republic of Korea; yoosa1224@naver.com

**Keywords:** bispectral index, cardiopulmonary bypass, sevoflurane

## Abstract

**Background/Objectives**: Volatile anesthetic dosing during cardiopulmonary bypass (CPB) is poorly standardized. We estimated the end-tidal sevoflurane (ETsevo) concentration required to maintain adequate anesthesia during CPB and investigated the effects of age and body temperature. **Methods**: This study is a PRISMA-compliant, PROSPERO-registered meta-analysis. PubMed, Embase, and the Cochrane Library were searched. Prospective studies of adults who underwent cardiac surgery with CPB and receiving sevoflurane were included. Primary outcome was mean ETsevo concentration when bispectral index (BIS) was 40–60. Three-level random-effects meta-analytic models with robust variance estimation were used to pool repeated measurements within studies. Age and body temperature were then examined as study-level moderators. Risk of bias was determined using ROBINS-I. **Results**: Five studies (*n* = 129) fulfilled the criteria. Pooled ETsevo during CPB was 0.88 vol% (95% confidence interval [CI] 0.29 to 1.46; *p* = 0.02) with substantial heterogeneity (I^2^ = 87.6%). Body temperature was not a significant moderator (difference 0.26 vol%; 95% CI −1.12 to 1.64; *p* = 0.27). Higher mean age was associated with lower ETsevo, evidenced by the finding that patients with a mean age of >62.0 years required 0.45 vol% less ETsevo (95% CI −0.78 to −0.13; *p* = 0.01), and sensitivity analysis revealed a 0.05 vol% decrease per additional year. **Conclusions**: To maintain BIS at 40–60 during CPB, the estimated ETsevo requirement is 0.88 vol% (minimum alveolar concentration 0.53–0.58 in patients in their 60s). Requirements decreased with age, and body temperature exerted no detectable effect.

## 1. Introduction

Since the introduction of cardiopulmonary bypass (CPB) in 1953, cardiac surgery has progressed dramatically, enabling increasingly complex procedures and improving perioperative outcomes [1]. Despite these advances, the anesthetic management during the maintenance phase of CPB remains poorly standardized [2]. In particular, the optimal concentration of volatile anesthetics required during CPB remains uncertain because of substantial physiological changes during bypass, such as hemodilution, nonpulsatile flow, absence of pulmonary gas exchange, and hypothermia.

Recent studies have demonstrated that electroencephalogram (EEG) monitoring-based anesthesia management is effective in improving surgical outcomes, minimizing intraoperative risks, evaluating anesthesia requirements, and reinforcing the fundamental role of anesthesia [3,4]. The efficacy of EEG-guided anesthesia in patients undergoing cardiovascular surgery has also been investigated, with findings demonstrating a reduction in the duration of respiratory support [2]. Nevertheless, no significant comparative advantage of EEG-guided anesthesia over standard practices was found in terms of postoperative delirium, length of hospital stays, length of ICU stays, mortality, or intraoperative blood transfusion requirements [5]. Furthermore, raw or processed EEG signals during CPB are frequently distorted by confounding factors such as hypothermia, pump flow, hemodilution, and nonphysiological cerebral perfusion [6]. Consequently, the reliability of EEG-based monitoring under these conditions has been questioned.

Accordingly, even with possible benefits, the inconsistency of data and the absence of a reliable method to titrate sevoflurane concentration during CPB have prevented the establishment of an evidence-based, universally accepted anesthetic protocol. Previous systematic reviews concluded that although volatile agents can be safely administered during CPB, the widespread adoption of such practice remains hindered by technical challenges, risk of anesthetic leakage, and lack of outcome-driven dosing guidelines [7,8].

Hence, the optimal sevoflurane concentration required during CPB remains unknown, and the available evidence does not support a clear superiority of certain concentration regimens in terms of outcome or safety. Therefore, we conducted a meta-analysis to determine the optimal sevoflurane concentration required for maintaining anesthesia during CPB.

## 2. Materials and Methods

### 2.1. Search Strategy

This meta-analysis was conducted according to the Preferred Reporting Items for Systematic Reviews and Meta-Analyses guidelines and was registered in the PROSPERO database (ID CRD420251229616). Two independent investigators (S.-H.L. and K.K.) systematically searched PubMed, Embase, and the Cochrane Library for relevant studies published up to 19 November 2025. The combination of search terms included “cardiopulmonary bypass,” “sevoflurane,” and “bispectral index.”

### 2.2. Study Selection Criteria and Data Collection

This meta-analysis included studies that fulfilled the following eligibility criteria: adult patients (aged ≥ 18 years) who underwent cardiac surgery with CPB under inhalational anesthesia. Articles not published in English were excluded. No restrictions were placed on the publication date. Both randomized controlled trials and observational studies were eligible for inclusion. Two reviewers (S.-H.L. and K.K.) independently conducted full-text screening and data extraction.

### 2.3. Data Items and Risk-of-Bias Assessment

The extracted variables included patient age, sample size, body temperature (°C), expiratory sevoflurane concentration (vol%) measured as end-tidal values (when unavailable, inspiratory concentrations were collected), and bispectral index (BIS) values and were summarized as mean ± SD. When data were reported as 95% confidence intervals (CIs), standard deviations were derived according to the methods described in the Cochrane Handbook [9]. The overall mean ± SD of patient age and median (range) of temperature were calculated using MATLAB R2025b (MathWorks, Inc., Natick, MA, USA). We summarized temperature at the study level by averaging all reported temperature values within each study to account for repeated temperature measurements within individual studies [10].

The risk of bias of the included studies was determined using the Cochrane Risk of Bias in Non-Randomized Studies—of Interventions (ROBINS-I) tool [11].

### 2.4. Effect Measures

The primary effect measure in this meta-analysis was the pooled mean end-tidal sevoflurane (ETsevo) concentration required to maintain BIS values within the target range of 40–60 during CPB. When only inspired sevoflurane concentrations were reported, these values were converted to ETsevo concentrations by multiplying the inspired concentration by a conversion ratio (ETsevo/inspired sevoflurane concentration) derived from a reference study [12] that provided paired inspired and end-tidal measurements. In one study, sevoflurane was administered at a fixed concentration of 1.8 vol% throughout the CPB period [13]. For that study, only the periods during which the mean BIS value remained within 40–60 were included in the primary effect measure.

Subgroup analyses were conducted to investigate whether the required ETsevo concentration differed according to body temperature or patient age.

### 2.5. Synthesis Methods

Several studies reported more than one effect size because sevoflurane concentrations were measured repeatedly at different CPB temperatures. To account for this, we used a three-level random-effects meta-analysis [14]. In this model, repeated measurements within a study were treated as level-2 effects, and studies were considered level-3 units, with sampling variance representing the level-1 component. Robust variance estimation was applied to provide stable standard errors, CIs, and *p* values, considering the limited number of studies and the presence of multiple effect sizes per study [15]. To summarize heterogeneity, we calculated multilevel I^2^ values, representing the proportion of total variation at each level [16].

The primary analysis focused on estimating the ETsevo concentration required during CPB. We also investigated whether patient age and body temperature during CPB were associated with this requirement. Because these variables were reported at the study level, they were treated as between-study moderators. For the main moderator analyses, age and body temperature were each entered separately as binary moderators, and their effects were estimated within the three-level meta-analytic model to determine statistical significance. Then, a sensitivity analysis was performed to further explore the impact of age on sevoflurane requirements, in which mean age was considered a continuous, centered variable rather than a binary moderator [17]. Certainty of evidence was determined using GRADE evaluation [18].

All statistical analyses were conducted using R version 4.5.2 (R Foundation for Statistical Computing, Vienna, Austria) with the metafor and clubSandwich packages. A three-level mixed-effects model was fitted using the rma.mv() function with restricted maximum likelihood estimation. Statistical significance was defined as *p* < 0.05.

## 3. Results

### 3.1. Study Selection

**Figure 1:**
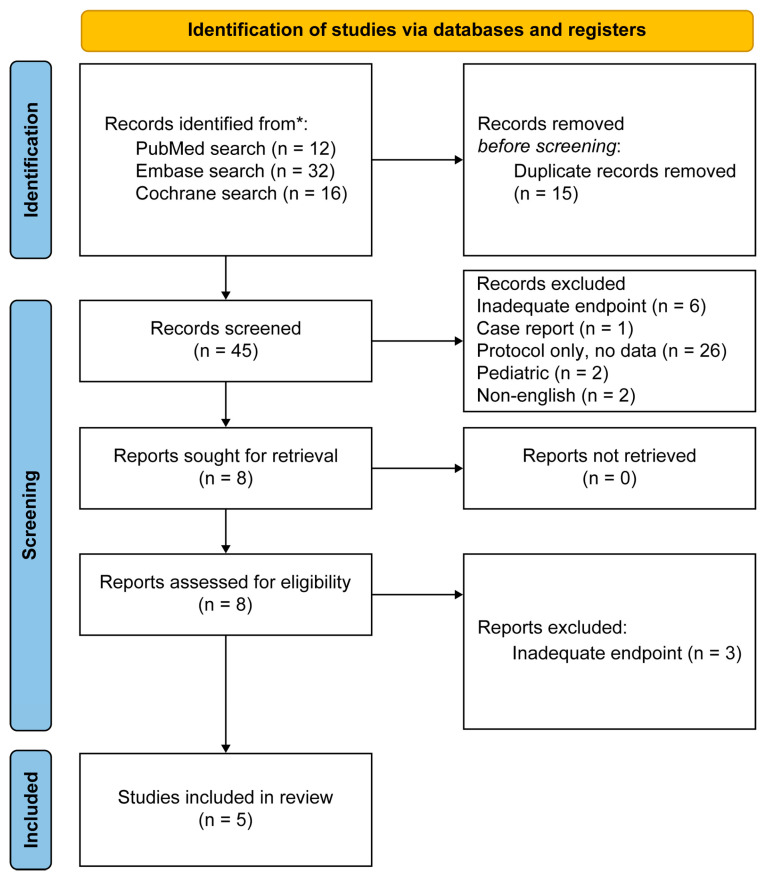
Preferred reporting items for systematic reviews and meta-analyses flow diagram.

The database search identified 60 articles from PubMed, Embase, and the Cochrane Library. After removing 15 duplicates, 45 records remained for screening the title and abstract. Eight articles were then reviewed in full, and five prospective studies fulfilled the eligibility criteria. Three articles were excluded after full-text evaluation because their endpoints did not match the requirements of this meta-analysis (Figure 1).

A total of 60 records were identified from database searches. After removing duplicates, 45 records were screened. Moreover, 37 records were excluded for the following reasons: inadequate endpoint (*n* = 6), case report (*n* = 1), protocol only with no data (*n* = 26), pediatric population (*n* = 2), and non-English publication (*n* = 2). Eight full-text reports were sought, and all were retrieved. The eight reports were evaluated for eligibility, of which three were excluded due to inadequate endpoints. Five studies were included in the final review (Table 1).

### 3.2. Study Characteristics

This meta-analysis included five prospective studies, with sample sizes ranging from 9 to 50 patients [12,13,19,20,21]. The mean age of participants was 53.3–67.0 years, and there were 129 patients. Most studies enrolled patients who underwent coronary artery bypass grafting, although some included individuals who underwent mitral valve replacement, aortic valve replacement, or mixed elective cardiac operations. The mean ± standard deviation age of participants in the five studies was 62.0 ± 9.59 years, and the median (range) body temperature during CPB was 32.9 (31.0 to 37.0) °C.

Three studies have reported ETsevo concentrations [13,19,21], one study has reported only inspired sevoflurane concentration [20], and another study has reported both inspired sevoflurane and ETsevo concentrations [12]. Inspired sevoflurane concentration was converted to ETsevo concentration as described in the Methods section.

Sevoflurane administration methods differed across studies. Three studies titrated ETsevo concentrations to maintain BIS targets between 40 and 60 [12,19,20]. One study adjusted inspiratory sevoflurane concentrations to achieve BIS values of 40–50 [21], and another study administered a constant inspiratory sevoflurane concentration of 1.8 vol% throughout the CPB procedure [13].

All studies reported body temperature during CPB, sevoflurane concentrations (end-tidal or inspiratory), and BIS measurements, which were used for the pooled analyses of sevoflurane requirements.

### 3.3. Risk of Bias

**Figure 2:**
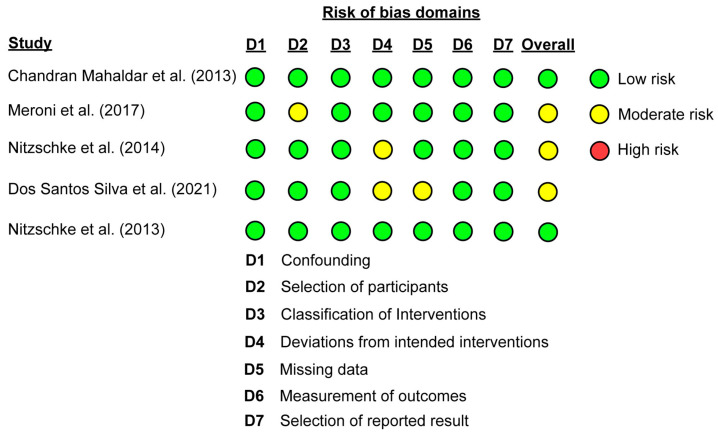
Risk-of-bias assessment of the included studies based on the Risk of Bias in Non-Randomized Studies—of Interventions (ROBINS-I) tool. Chandran Mahaldar et al. (2013) [19], Meroni et al. (2017) [12], Nitzschke et al. (2014) [20], Dos Santos Silva et al. (2021) [21], and Nitzschke et al. (2013) [13].

Figure 2 summarizes the risk-of-bias assessment for each study. In the ROBINS-I evaluation, one study reported some concerns related to participant selection, two studies presented some concerns regarding the classification of interventions, and one study presented some concerns due to missing data. Overall, three studies were judged to have a “moderate” risk of bias, and the remaining two studies were considered to have a “low” risk of bias.

Colored circles indicate domain-level judgments (green, low risk; yellow, moderate risk; red, high risk), and the overall risk-of-bias rating for each study is depicted in the final column. No study was judged to have a high risk of bias.

### 3.4. Overall Analysis

The findings from the three-level mixed-effects model are presented in Table 2. The pooled estimated ETsevo concentration required to maintain BIS values within 40–60 during CPB was 0.88 vol% (95% CI, 0.29 to 1.46; *p* = 0.02). Considerable heterogeneity was found (I^2^ = 87.6%).

### 3.5. Effect of Body Temperature and Age

Body temperature and age were examined as moderators in separate three-level mixed-effects models. Age was dichotomized at its mean value, and body temperature was dichotomized as its median value. The moderator effect was defined as the difference in estimated ETsevo concentration between the lower and higher categories.

Body temperature exerted no statistically significant impact on the required ETsevo concentration. The estimated moderator effect was 0.26 vol% (95% CI, −1.12 to 1.64; *p* = 0.27), and heterogeneity remained high (I^2^ = 88.6%, Table 3).

**Figure 3:**
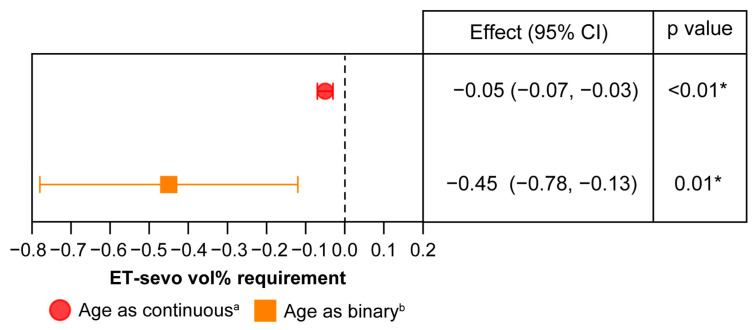
Sensitivity analysis and effect of age (continuous and binary) on end-tidal sevoflurane requirement. ET-sevo, end-tidal sevoflurane; CI, confidence interval. ^a^ Required ET-sevo decreased by 0.05 vol% per 1-year increase in age. ^b^ Required ET-sevo was 0.45% lower in patients aged ≥62.0 years than in those aged <62.0 years. * Statistically significant.

In the three-level mixed-effects model, age exerted a significant moderating effect. Studies with a median patient age > 62.0 years required a lower sevoflurane concentration during CPB than those with patients less than this age threshold. The estimated difference was −0.45 vol% (95% CI, −0.78 to −0.13; *p* = 0.01), and the heterogeneity decreased (I^2^ = 75.4%, Table 3). Furthermore, the sensitivity analysis revealed that the required sevoflurane concentration decreased by 0.05 vol% per 1-year increase in age (Figure 3).

## 4. Discussion

This meta-analysis revealed that the estimated optimal ETsevo concentration required during CPB was 0.88 vol%. Increasing age was associated with a statistically significant reduction in sevoflurane requirement, whereas higher body temperature tended to increase the requirement, although this effect was not statistically significant.

In cardiac surgery, anesthesiologists play a crucial role in optimizing anesthetic depth and physiological stability during CPB by titrating volatile anesthetics in the setting of altered pharmacokinetics and pharmacodynamics, thereby maintaining hemodynamic stability and organ perfusion and preventing intraoperative awareness [2]. Therefore, several previous studies have described EEG-guided anesthetic maintenance [19,20,21]; however, no significant differences were observed in postoperative outcomes [5]. Furthermore, a recent review reported that despite the effects of hemodilution, hypothermia, alterations in blood flow distribution, and hypotension, as well as the accumulation of anesthetics within the oxygenator and CPB circuit during CPB, ETsevo concentrations reliably reflect plasma sevoflurane levels, thereby allowing adequate and safe anesthesia to be maintained with relatively lower anesthetic requirements during CPB [22]. Therefore, to identify the optimal sevoflurane concentration required during CPB, we conducted this meta-analysis of existing studies that evaluated ETsevo concentrations during CPB.

When converted into minimum alveolar concentration (MAC) values for patients in their 60s, an ETsevo concentration of 0.88 vol% corresponds to approximately 0.53–0.58 MAC [23]. This value is lower than the mean anesthetic concentration of 0.79 MAC reported in a large randomized clinical study involving patients aged ≥60 years who underwent cardiac surgery [5]. In that study, EEG monitoring guided the inhalational anesthetic dosing. These findings align with those of previous studies that demonstrated reduced sevoflurane requirements for inhalational anesthetics during the CPB period [22].

Hypothermia has been consistently associated with reduced requirements for volatile anesthetics in previous studies [24,25]. However, in this meta-analysis, patient body temperature during CPB was not a significant determinant of sevoflurane requirement. We considered two plausible explanations. First, blood pressure may affect BIS-guided titration of anesthetic depth. BIS values can decrease when the mean arterial pressure decreases to less than the lower limit of cerebral autoregulation [26]. This phenomenon may result in unnecessary reductions in volatile anesthetic delivery. Nevertheless, across the studies included in this meta-analysis, the mean arterial pressure during CPB was generally maintained between 50 and 80 mmHg. This range is generally considered sufficient to preserve cerebral autoregulation, which probably limited the impact of blood pressure–related confounding. Second, the degree of hypothermia in the included studies was relatively modest. Marked reductions in EEG-derived depth indices are typically observed during deep hypothermia, generally at temperatures of approximately ≤27 °C [27]. In contrast, the lowest body temperature reported in the present dataset was 29 °C. Within this narrow and mild hypothermic range, any temperature-related reduction in sevoflurane requirement may have been too small to be detected.

We next evaluated the certainty of evidence to provide a transparent appraisal of the reliability of the effect estimates, considering the heterogeneity and methodological limitations of the included studies. The result of the certainty of evidence is shown in Table S1. The certainty of evidence for the pooled mean sevoflurane requirement was rated as very low, primarily due to the severe risk of bias, high heterogeneity (I^2^ = 83.7%), and imprecision of the estimate. The certainty for the temperature effect (low vs. high body temperature) was also very low because the CI was wide and compatible with both clinically important increases and decreases in sevoflurane requirements. However, the evidence for an inverse association between age and sevoflurane requirement was judged as low certainty but consistent and precise estimates across sensitivity analyses.

### Limitation

Several limitations of this meta-analysis must be acknowledged. First, although we used the ROBINS-I tool to determine the risk of bias in the included studies, the potential for residual confounding and methodological bias remains a limitation. Furthermore, despite the low certainty of evidence for the pooled sevoflurane requirement and low certainty for the age effect, the consistency and precision of the effect estimates suggest a reasonable association.

Second, anesthetic management was not standardized across the included studies. Co-administered sedatives (e.g., propofol and midazolam) and opioids (e.g., fentanyl, sufentanil, and remifentanil) can impact BIS values and volatile anesthetic requirements, even when administered prior to CPB. Because dosing regimens, timing relative to CPB, and the use of adjunctive agents were variably reported, adjustment for these factors was not feasible, likely contributing to between-study heterogeneity.

Third, ETsevo concentrations were not consistently reported, and one study provided only inspired concentrations. Although inspired values were converted to estimated end-tidal concentrations using a ratio derived from a reference study with paired measurements, this approach introduces additional uncertainty. The inspired-to-end-tidal relationship may vary with fresh gas flow [28], circuit dynamics [29], and CPB-related gas exchange conditions [30]. Furthermore, oxygenator type [31] may influence blood and alveolar concentrations and BIS responses. These technical factors were not uniformly described and therefore could not be modeled directly.

Fourth, sampling time points were not consistently specified. Some studies linked sevoflurane and BIS data to elapsed time after CPB initiation, while others reported only a single temperature, and most did not clearly distinguish CPB phases (cooling, steady-state hypothermia, and rewarming). Because volatile anesthetic requirements and BIS behavior may vary across CPB phases and temperatures, incomplete phase-specific reporting limits the generalizability of our findings. To enable more reliable interpretation, future studies should standardize and report ETsevo measurements, fresh gas flow, vaporizer and oxygenator settings, and phase-specific sampling time points (cooling, steady-state hypothermia, and rewarming).

## 5. Conclusions

Our meta-analysis suggested that the optimal ETsevo concentration required during CPB was 0.88 vol%. When converted into MAC values for patients in their 60s, it corresponds to approximately 0.53–0.58 MAC. Considering the lack of evidence-based guidelines for anesthetic management during CPB, there exists a need for precisely designed studies integrating pharmacokinetic/pharmacodynamic monitoring, cerebral perfusion assessment, and postoperative outcome measures. Therefore, the results of this meta-analysis may provide clinically relevant insights to inform anesthetic dosing during the CPB period.

## Figures and Tables

**Table 1:** Study characteristics.

Study	Patients	Age (Years)	Surgery	Sevoflurane Administration Method	Data
Chandran Mahaldar et al. (2013) [19]	50	58.5 (7.3)	CABG	End-tidal sevo titrated to maintain BIS 40–50	Body temperature, end-tidal sevo concentration, and BIS
Meroni et al. (2017) [12]	10	53.3 (10.5)	MVR	End-tidal sevo titrated to maintain BIS 40–60	Body temperature, end-tidal sevo concentration, and BIS
Nitzschke et al. (2014) [20]	31	67.0 (9.8)	CABG, AVR, or combined	Inspiratory sevo adjusted to maintain BIS 40–60	Body temperature, inspiratory sevo concentration, and BIS
Dos Santos Silva et al. (2021) [21]	9	61.5 (6.9)	CABG	End-tidal sevo dosed to determine ED50 and ED90 (BIS 40–50)	Body temperature, ED50 of end-tidal sevo concentration, and BIS
Nitzschke et al. (2013) [13]	29	65.7 (9.2)	Elective cardiac surgery	Constant inspiratory sevo (1.8 vol%)	Body temperature, end-tidal sevo concentration, and BIS

CABG, coronary artery bypass grafting; MVR, mitral valve replacement; AVR, aortic valve replacement; sevo, sevoflurane; BIS, bispectral index; ED, effective dose. Age is reported as mean (standard deviation).

**Table 2:** Three-level meta-analytic estimate of end-tidal sevoflurane concentration during cardiopulmonary bypass.

Outcome	Estimated ETsevo, vol%	95% CI	*p*	Heterogeneity I^2^, %
End-tidal sevoflurane concentration	0.88	0.29–1.46	0.02 *	87.6

ETsevo, end-tidal sevoflurane; CI, confidence interval. * Statistically significant.

**Table 3:** Three-level meta-analytic estimate of end-tidal sevoflurane concentration during cardiopulmonary bypass according to moderator variables.

Moderator	Estimated ETsevo, vol% ^a^	95% CI	Moderator Effect, vol% ^b^	Moderator 95% CI	*p* for Moderator	Heterogeneity I^2^, %
Body temperature ^c^	0.70	0.36, 1.05	0.26	−1.12 to 1.64	0.27	88.6
Age ^d^	1.14	0.89, 1.38	−0.45	−0.78 to −0.13	0.01 *	75.4

ETsevo, end-tidal sevoflurane; CI, confidence interval. ^a^ Estimated ETsevo concentration in the reference group (body temperature ≤ 32.9 °C and patient age ≤ 62.0 years). ^b^ Positive values indicate a higher ETsevo requirement in the comparison group, and negative values indicate a lower requirement relative to the reference group. ^c^ Body temperature during cardiopulmonary bypass was dichotomized as ≤32.9 °C (reference group) and >32.9 °C. ^d^ Age was dichotomized at the study-level median as ≤62.0 years (reference group) and >62.0 years. * Statistically significant.

## Data Availability

The datasets generated and/or analyzed in the current study are available from the corresponding author upon reasonable request.

## Supplementary Materials

The following supporting information can be downloaded at: https://www.mdpi.com/article/10.3390/biomedicines14030535/s1, Table S1: Grading of recommendations assessment, development, and evaluation for accessed outcomes.

## Abbreviations

The following abbreviations are used in this manuscript:

BIS	Bispectral index
CPB	Cardiopulmonary bypass
CI	Confidence interval
EEG	Electroencephalogram
ETsevo	End-tidal sevoflurane
MAC	Minimum alveolar concentration
ROBINS-I	Risk of Bias in Non-Randomized Studies—of Interventions
