# Supplementary material for: Estimated End-Tidal Sevoflurane Concentration to Maintain Optimal Anesthetic Depth During Cardiopulmonary Bypass: A Meta-Analysis
Source: Biomedicines. 2026 Feb 27;14(3):535. doi: 10.3390/biomedicines14030535 (PMC13023477; doi:10.3390/biomedicines14030535)
Supplement: Supplementary file 1 [file biomedicines-14-00535-s001.zip › biomedicines-4174166-supplementary.pdf]

Table S1. Grading of recommendations assessment, development, and evaluation for accessed outcomes.

Certainty assessment						
**Participants**	**Risk of bias**	**Inconsistency**	**Indirectness**	**Imprecision**	**Publication bias**	**Overall certainty of evidence**
Overall outcome						
129 (5 studies)	Serious[a]	Serious[b]	Not serious	Not serious	Unclear	Very low
Body temperature						
129 (5 studies)	Serious[a]	Serious[b]	Not serious	Very serious[c]	Unclear	Very low
Age						
129 (5 studies)	Serious[a]	Not serious	Not serious	Not serious	Unclear	Low

a. Among the five studies that evaluated this outcome, three were classified as having some concerns regarding the risk of bias.

b. High heterogeneity of the point estimate of outcome was observed.

c. The 95% confidence interval was wide (−1.12 to 1.64), indicating very serious imprecision.
